# ‘It’s like tumbleweeds everywhere’: An Interpretative Phenomenological Analysis of the lived experience of being diagnosed with and living with narcolepsy

**DOI:** 10.1177/13591053231221373

**Published:** 2024-01-29

**Authors:** Bella Kilmartin, William Day

**Affiliations:** Aston University, UK

**Keywords:** chronic illness, diagnosis, experience, Interpretative Phenomenological Analysis (IPA), narcolepsy, qualitative methods

## Abstract

There is a lack of awareness of how sleep health and sleep disorders are experienced. Previous research has found that living with narcolepsy has a debilitating impact on several areas of an individual’s life alongside significant diagnostic delays. This study uses a phenomenological, qualitative methodology to explore experiences of being diagnosed with and living with narcolepsy. Six women with type 1 narcolepsy participated in semi-structured interviews. Transcripts were analysed using Interpretative Phenomenological Analysis. Capturing the whole illness experience of narcolepsy, our analysis illuminated three superordinate themes; ‘minimising, dismissing and downplaying symptoms’, ‘navigating the winding journey to diagnosis’ and ‘a different way of living’. Through our analysis, we are able to demonstrate the affective impact lack of awareness of sleep and sleep disorders has; resulting in significant diagnostic delays and a lack of support post-diagnosis. Findings demonstrate a need for greater awareness and increased support.

## Introduction

Narcolepsy is an under-researched chronic neurological disorder marked by sleep-wake dysregulation (Bassetti et al., 2019). It is primarily characterised by excessive daytime sleepiness (EDS) and intrusions of rapid eye movement (REM) sleep into wakefulness (Nishino, 2007). The cause of narcolepsy is the loss of over 90% of neurons in the hypothalamus that produce hypocretin (Mahlios et al., 2013). Hypocretin is a neuropeptide that regulates sleep-wakefulness (Savvidou et al., 2013).

There are two types of narcolepsy: Type 1 (narcolepsy with cataplexy) and Type 2 (narcolepsy without cataplexy). Narcolepsy is classified as a rare disease that affects approximately one in 2000 people in the United States and Europe (Mahoney et al., 2019).

### Diagnosing narcolepsy

An important characteristic of narcolepsy is the long diagnostic delays that are similarly seen in other rare and chronic illnesses (Khatami et al., 2016). Diagnostic delays refer to the delay between the first onset of symptoms and receiving a correct diagnosis (Morrish et al., 2004). For narcolepsy, this time ranges from 1 to 60 years (Parkes et al., 1998), with the average time ranging from 9 to 12 years (Frauscher et al., 2013; Ohayon et al., 2021). A significant contributor to diagnostic delay in narcolepsy is poor knowledge of sleep disorders among general practitioners (Hassed et al., 2012). Another contributing factor appears to be the delay from symptom onset to first consultation and the delay from consultation to diagnosis, with an average of 8.2 and 3.6 years, respectively (Ohayon et al., 2021).

Diagnostic delays are significant as individuals with undetected or undiagnosed narcolepsy face an increased risk of disease linked to issues with sleep. Furthermore, they also face an inability to keep up with peers and a lack of understanding from individuals around them (Flygare and Parthasarathy, 2015). Diagnostic delay can additionally lead to patients receiving incorrect diagnoses and medication, delaying access to the right treatment and exacerbating symptoms’ impact (Maski et al., 2017). However, no research has explored the patient’s experience of this.

### Living with narcolepsy: A chronic health condition

A large proportion of narcolepsy research has followed the biomedical model of illness. The biomedical model of illness focuses solely on the biology of a condition without considering any psychosocial or environmental influences (Engel, 1977). Research has predominantly focused on finding a cause of narcolepsy, which was discovered as recently as 1999 (e.g. Lin et al., 1999). Since then, a large proportion of research has focused on physical symptoms and finding adequate treatments. However, despite advancements in the pharmacological management of narcolepsy symptoms, Maski et al. (2017) found that 80.1% of participants indicated that their symptoms were either the same since diagnosis or worse and more unpredictable.

Living with narcolepsy can be debilitating, impacting individuals emotionally, financially and socially (Dodel et al., 2007). Teixeira et al. (2004) suggest that the impact of narcolepsy on health-related quality of life is equivalent to or worse than the impact of Parkinson’s disease or epilepsy. EDS significantly interferes with daily living and restricts the number of leisure activities participants can engage in (Dodel et al., 2007; Teixeira et al., 2004). When assessing psychosocial issues, narcolepsy impacts education, work, relationships, activities of daily living and leisure activities (Goswami, 2016).

Qualitative research on narcolepsy appears to be limited to a focus on how growing up with narcolepsy during adolescence has been found to affect all aspects of everyday life and well-being (Wehrle and Bruck, 2011) or to a focus on specific symptoms such as automatic behaviour (Morandin and Bruck, 2013) and cataplexy (Franceschini et al., 2020). While previous qualitative research into narcolepsy sought to take a phenomenological approach, little detail was given on the methods used: rather than a rigorous process of analysis, analytical insights were simply grouped into themes or categories. Additionally, there is a lack of research that explores the whole illness experience. The illness experience of how the condition is *as lived* and how individuals make sense of the condition. It involves gaining insight into their beliefs and how they perceive their chronic illness (Larsen, 2014).

An alternative to the biomedical model that helps to understand the illness experience is the illness-constellation model. This model views illness as an experience that affects the individual and their significant others in four stages (Morse and Johnson, 1991). The first stage is known as the stage of uncertainty (where individuals notice symptoms but do not know what is wrong), the stage of disruption (they decide that the illness is real and seek help), the stage of striving to regain self (trying to make sense of the illness) and the stage of regaining wellness (adjusting and accepting a new level of functioning). These stages have been evidenced in the illness experience of those with aphasia and neurogenic-related language disorders, particularly in terms of trying to make sense of the illness (LaPointe, 2011). This model could provide a greater understanding of the whole experience of narcolepsy, from diagnosis to management, as diagnostic delays appear to be an influential factor in how individuals experience a condition. Informed by the illness-constellation model, this study aims to understand and capture the whole illness experience of being diagnosed with and living with narcolepsy.

## Method

### Design and participants

A phenomenological approach was taken to focus on the experience of narcolepsy from the individual’s perspective (Nelson, 2011). Purposive homogeneous sampling was used to recruit a homogeneous sample size of between four and six participants (Smith et al., 2009). As narcolepsy is considered rare, researchers decided that having a diagnosis of narcolepsy was sufficient to define the criteria of the sample. Recruitment was done by advertising the study on the author’s personal narcolepsy awareness Instagram page, providing access to people with narcolepsy. Participants responded to the advertisement through private messages or email. There were more responses than needed, so participants were recruited on a first-come, first-served basis to avoid bias.

The final sample consisted of six women aged between 18 and 34. Four of the women were from the UK, and two of the women were from the USA. Although the difference in participants’ nationalities might be a threat to homogeneity, it was decided that, as they were all women with type 1 narcolepsy, the sample was sufficient to explore a shared perspective on narcolepsy. The average time from participants’ first experiencing symptoms to diagnosis was 13 years.

### Ethics and data collection

Ethical approval was granted by Aston University’s School of Psychology Protocol and Ethics Review Board. Data was collected using semi-structured one-to-one interviews to gain a first-person account of narcolepsy in which participants primarily control the conversation’s direction. Open-ended questions were asked to encourage conversation, such as ‘*Could you tell me about when you first started developing symptoms of narcolepsy?*’. Six virtual interviews were conducted via Microsoft Teams. Participants gave informed consent before the interview took place, and participants were reminded of their right to withdraw and their right to decline to answer any questions during the interview. All interviews lasted between 40 and 60 minutes. Interviews were audio-recorded and transcribed verbatim. To maintain anonymity, participants were given pseudonyms that reflected their gender and ethnicity.

### Analysis

Transcripts were analysed using Interpretative Phenomenological Analysis (IPA). As the analytic work was undertaken prior to the publication of Smith et al. (2022), analysis was completed using the original processes and terms as outlined by Smith et al. (2009). The analytical ‘goal’ was to explore and gain a deeper understanding of how individuals make sense of their lived experiences through their accounts of being diagnosed with and living with narcolepsy (Gill, 2015). This understanding was gained through a phenomenological and interpretative approach by giving participants a voice and then making sense of their experiential claims from a psychological perspective (Larkin et al., 2006). As IPA has an idiographic approach, the process starts with an inductive analysis of individual cases before moving on to cross-case analysis (Smith, 2004). Analysis involved moving forward and backwards between the data to gain better focus and understanding (Smith and Firth, 2011). It was important to repeatedly refer to the transcripts and ensure the analysis was grounded in the data, as the first author is someone living with narcolepsy. Consequently, the researcher kept a reflective journal to record a trail of biases and thought processes, which were discussed with the second researcher throughout.

## Results

Our analysis focused on three superordinate themes composed of seven subordinate themes Table 1 shows the structure of this analysis.

**Table 1.** Analytical findings.

Superordinate themes	Subordinate themes	Description
Minimising, dismissing and downplaying symptoms	Not noticing symptoms at the time	Explores how participants experienced symptoms before they noticed them as they often minimised them and how even after, they still downplayed the impact of symptoms
	Downplaying symptoms after diagnosis	
Navigating the journey to diagnosis	Noticing and acknowledging something is wrong	Participants explore their experiences of the journey up to being diagnosed with narcolepsy. Participants seemed to make sense of this in two distinct parts.
	The passive to active search for answers	
A different way of living	Narcolepsy takes control of day-to-day life	Focuses on the individuals lives post diagnosis, the restriction that narcolepsy places on their lives and the lack of support that they have.
	Feeling different socially and physically	
	Lack of support	

## Minimising, dismissing and downplaying symptoms

### Not noticing symptoms at the time

All participants noted that excessive daytime sleepiness was the first symptom that they experienced. However, at the time, while they did become aware of their sleepiness, they did not consider it to be a major cause for concern or a symptom. Bethany, with her diagnosis and knowledge of narcolepsy, could now retrospectively understand that her coping mechanisms at the time were actually ‘symptoms’. This experience was similar across all participants; when asked to reflect, they realised that their first experience of narcolepsy symptoms was earlier than they originally thought.

*And looking back, I see all these changes of how I was trying to live my life the same. . .. Looking back, I did not see- I didn’t think anything of this at the time. But [. . .] I worked as a phlebotomist in health care and I had worked like 12 hour shifts. And I was now working different hours. Much less stress. And it was only like, I think, eight hour shifts. But I was drinking 1 to 2 cranberry red bulls and sometimes a diet Dr. Pepper to get through a shift. And looking back, that is insane*

- ***Bethany***

Being asked to reflect on their first experience of symptoms allowed the participants to explore their sense-making of the sleepiness when they were first experiencing it. Katie and Laura’s symptoms were understood through an intersubjective comparison, considering how their experiences of sleepiness differed from others around them. The sleepiness was dismissed by their friends and family, who considered it something that would happen to everyone. These dismissals overrode participants’ own feelings about their sleepiness, despite coming from people who were not the ones experiencing the sleepiness. Katie and Laura internalised these perceptions from other people, which played a pivotal role in their own dismissal of their symptoms. As a result, they did not notice the sleepiness as a symptom of a greater problem.

*I didn’t really think about it. I was just like, I’m really tired. And my mum, like and my mum and dad’s friends and things like, they’ll just be like, “oh well, I was really tired when I was growing up too. So that must be what it is*

**-*Laura***

The use of ‘casual’ phrases such as ‘*oh well*’ when recalling how their family described their experiences draws attention to the minimisation of their sleepiness. The dismissive phrase ‘oh well’ promotes an acceptance that their sleepiness is normal and nothing of concern. This was further solidified for Katie when her sleepiness was dismissed by medical professionals as ‘**probably**. . . hormonal changes’.

For others, rather than dismissing their sleepiness as something that everyone experiences, Abby and Alex thought of their sleepiness as a unique experience to them. Abby and Alex were perceived, and perceived themselves as, ‘sleepy’ and ‘lazy’. The use of these adjectives shows how their sleepiness was considered to be a trait of theirs rather than something that was happening to them. The implication was that there is nothing that can be done about their sleepiness; it is part of who they are, which enables it to be dismissed and not taken seriously. This even went as far as the sleepiness being considered a joke by Abby and Alex’s family and friends.

*It was always just kind of a bit of a joke. Like Abby goes for a nap after school, like she’s sleepy or whatever*

**-*Abby***

Although achieved in different ways, this subtheme highlights how all participants’ sleepiness was dismissed by other people, which they then internalised. This resulted in participants accepting the sleepiness as a normal experience for them.

### Downplaying symptoms after diagnosis

The internalisation of dismissals of their sleepiness impacted the way that participants felt about their symptoms even after receiving a diagnosis. However, there appears to be a subtle shift from dismissing symptoms to downplaying the impact they have. When discussing the difficulty she had in coming to terms with her narcolepsy diagnosis, Abby very quickly shuts this down and downplays her symptoms and the struggle by comparing her situation to others. Similarly, Alex downplayed the struggle by comparing it with cancer. Doing so acts as a ‘self-invalidation’ of their own challenges and embodied sensations:

*At the same time, you know, being 18 and being told, oh yeah, you’ve got an incurable neurological disorder, it’s a bit of a shock to the system. But that took me quite a little bit to deal with. Um, you know, it could be worse. I could have something, you know, like life threatening, which obviously narcolepsy isn’t but it’s still a difficult thing to hear*.

**-*Abby***

Participants are conflicted between acknowledging the difficulty of their symptoms and considering their struggles are unworthy of expression and unreasonable to complain about. As a result, they often downplay the impact of their symptoms. Talking about the reality of the impact of her symptoms left Katie feeling like she was exaggerating despite having significant struggles. This even went as far as Katie feeling guilty for accepting and seeking help for her disability and chronic neurological disorder that she needs and is entitled to:

*like I applied for PIP [personal independence payment] the other day and I almost felt guilty for applying for it because I was like I am able. So a lot of the questions and things they ask you is about how able you are. And that was when I then had to question how I able am I? so some of the questions are about preparing food. I always cook for myself like I’m independent. I’ll cook my dinners, but then I’ll have to consider, like I said, you about cutting myself before. Yeah, if I’m tired. I can’t go and do that because I’ll hurt myself. Or if I’m going to put something in the oven, it’s an hour. I need to make sure to stay awake for the hour because I have left things in there before and nearly burnt the house down. So I need to think about that. It almost makes you feel like you’re milking it*’

**-*Katie***

Other people’s perceptions of their symptoms were important in how they made sense of their symptoms. Therefore, when it was dismissed by others and not taken seriously, it resulted in participants failing to consider their sleepiness as a problem which was a significant contributor to diagnostic delays. The fact that these dismissals occur even after receiving a diagnosis, highlights how symptoms of narcolepsy are not taken seriously.

## Navigating the winding journey to diagnosis

### Realisation that something is wrong

The first step in the diagnosis journey for all participants began with noticing that there was something wrong. Abby’s sleepiness first started in primary school, yet she did not realise that something was wrong until she was in college. This is a substantial amount of time between her first experience of symptoms and considering that something was wrong. It was only upon her first experiences of cataplexy that she came to this realisation, and only then was there concern about the sleepiness after having experienced it for years.

*But then when I started college, erm, my cataplexy started, so I started to like my head would fall when I laughed erm and I started to have the full collapses. . . I was sleepy, but it wasn’t any point of concern until… after we realised about the cataplexy*

**-*Abby***

Similar to Abby, the realisation that something was wrong with participants came after experiencing sleepiness for a significant amount of time. For Alex and Laura, though, this realisation came after becoming increasingly frustrated when their sleepiness started occurring in environments that were not conducive to sleep. The sleepiness began disrupting their social and academic lives, which Alex and Laura found embarrassing. These feelings of embarrassment were the real trigger for them to address their sleepiness and consider it a problem.

*It became a bit of a joke. And I think the jokes were starting to get to me. And I was like, Mom, like, everyone’s teasing me. I don’t want another photo of me, like, drooling on the school, you know, like, fast asleep*

**-*Alex***

### The passive to active search for answers

In the search for answers, Brooke went through an iterative process of going back and forth to the doctors. This repetition resulted in her receiving incorrect diagnoses along the way and being referred to the wrong departments. It was a frustrating time for Brooke, as so many doctors insisted that they had an explanation for her symptoms. This resulted in Brooke being unable to trust her own body, and she lost faith in being able to find an answer for her symptoms. Even when her doctor suggested narcolepsy, she was reserved about believing it:

*I think if we treat your sleep disorder, all of these other things are going to go away. And I was like ha ha No, that’s great. I’ve been told that things were going to fix things my entire life, right? I was like, Whatever. I was like, okay, cool. Like that would be that would be great. I would love that, but it’s not going to happen. And then like when it started happening, I didn’t even know how to process it. And it was just I had become complacent in my misdiagnosis to the point that when I actually got diagnosed, like I, I was so sceptical and so reserved about believing it and and taking it in*’

**-*Brooke***

When compared to Brooke’s experience, we see that Abby took much more of an active role in her search for answers. Similar to Brooke, Abby was misdiagnosed, but she did not believe her diagnosis of functional neurological disorder made sense for her symptoms. Rather than relying on doctors to be the investigators, Abby took on this role herself. Through this, she learned about narcolepsy, which made her symptoms make sense, and she described how she intuitively knew this was what she had. After researching online, she came across narcolepsy, and this helped their symptoms make sense. This was the case for all participants; once they learned about narcolepsy, they had the intuition that they knew this was what they had. However, despite this intuition, Abby still faced being dismissed again by doctors through a display of medical power.

*And the most frustrating thing was. I like… Your own intuition. Like I, at that point, I was like, I knew it was narcolepsy. And he kept saying, no, it’s not. No, it’s not. And he wouldn’t order the right tests and he wouldn’t let me go to sleep clinic so for the next of like two years. I was like, I know this is what I’ve got. And he was just kind of holding it back*.

**-*Abby***

The process of searching for answers was a difficult and frustrating time for participants. Participants found searching for answers difficult because, while going through this process, they were made to feel ‘crazy’. They faced not being believed, being accused of making it up, and not being taken seriously.

## A different way of living

### Narcolepsy takes control over day-to-day life

Although participants felt relief at diagnosis, all participants articulated the control that narcolepsy has over the way that they live their lives. Participants discussed the difficulty of their symptoms and how their sleepiness and fatigue leave them with limited energy. This limited energy restricts the amount that they can do, which means they always have to be aware of their limitations. Participants discussed how this means they must carefully consider how much energy tasks require. As a result, they are forced to make compromises in their lives, such as having to take a nap rather than spending more time with family. To allow others to really try to understand what the restrictions of narcolepsy feel like, Laura uses the metaphor of a phone battery:

*It’s like if you’ve got 20% charge on your phone, do you want to listen to music? and do something else? or do one thing if that means it lasts longer like?*

**-*Laura***

This metaphor helps Laura effectively communicate how her finite resources force her to make priorities and sacrifices in order to make her energy last longer. Like Laura, Bethany makes use of the metaphor of dominoes to describe how her limited energy places restrictions on her day-to-day life. Living with narcolepsy forces her to strategically manage her day-to-day life; carefully thinking out and planning each day is essential to weighing up the impact that any activity will have on the next. Without this strategic management, it can result in an unworkable ending:

*I recently had tried to start explaining it, like, not playing the game of dominoes, but like setting up dominoes to fall down. I feel like that’s how I have to think in my head all the time. Because if I don’t line up my dominoes correctly, the ending is not going to work*

**-*Bethany***

However, sometimes this consideration of energy limits is not always effective enough. While participants can do all they can to try to manage their symptoms, they often find that their symptoms are inescapable. Alex reflects on the differing ways in which narcolepsy controls her life. Rather than solely focusing on symptoms, Alex pushes the concept of narcolepsy’s control further. She articulates how, for her, living with narcolepsy leaves her feeling that she has no control over her life. In contrast to Laura and Bethany, who, through being attentive to their energy limits, are able to optimise the management of the condition, Alex feels that there is *nothing* she can do to regain that control over her life. Control and autonomy over her life have become irrevocably lost.

*So I think how I describe it to someone who doesn’t have it is I’d probably say that some of the words I’d use is like. . . loss of control. . .. It’s kind of like, obviously chronic illness that’s unexpected and can just leave you powerless in any situation. And as much as you can take medication and have naps, you’ll always be tired*

**-*Alex***

### Narcolepsy leaves you feeling different socially and physically

When considering what life with narcolepsy is like, some participants focused on the difference in their social lives. Abby and Laura compare their lives with other people their age and reflect on the fact that their lives look very different to others. Having narcolepsy meant that they often stayed at home rather than going out with friends. This resulted from narcolepsy forcing them to have different priorities than others. Abby really struggled with missing out on these shared experiences with friends; her life deviated from the social norms of people her age. This experience left Abby feeling isolated:

*I can’t just do something spontaneously and, and the difference in me in lifestyle because like they wanted to go out and party and drink and I was like, no, I want to stay home with a cup of tea in bed please (laughs). Yeah. So yeah, they were good in some aspects, but other aspects it was it was a bit difficult. Bit lonely*.

**-*Abby***

Bethany and Alex discuss how narcolepsy makes them feel physically different from others. It is these physical differences that prevent her from being able to do what others are doing. Bethany describes this as feeling like a turtle trying to keep up with others. The comparison of the turtle and the rabbit demonstrates this clear physical capability difference. However, she struggles to accept this difference and tries to not let this stop her from attempting to keep up with others. Alex, too struggles with the physical differences and does not like to be seen as having a weakness.

*so I’m still trying to be me, but I’m like, like, kind of like a turtle trying to keep up with rabbits. That’s how I feel*

**-*Bethany***

### Lack of support

Many participants discussed that after being diagnosed with narcolepsy, there was a lack of professional support. Their doctors did not provide them with any support apart from being prescribed medication. Participants left their appointment with a diagnosis of a life-changing chronic neurological disorder without any information about managing their condition or where they would be able to find support. This lack of support continued beyond the initial diagnosis and there was never any support put in place. Laura likens the lack of support to the cinematic metaphor of ‘tumbleweeds’; Laura was expecting and was waiting for support that never came. The use of tumbleweeds evokes feelings of desertion and abandonment by medical professionals:

*There was nothing. It was like, what’s it called? I don’t know. Like, you know, like in Texas movies where it’s just like really like tumbleweeds. That’s what it was like everywhere (laughs)*

**-*Laura***

This lack of support is significant as Bethany discusses the importance of feeling understood. Yet participants discussed that even if family and friends were supportive, they were unable to truly understand and appreciate the difficulties and challenges that they faced. As highlighted by Bethany, this led to participants having to seek out their own forms of support. Bethany, like many other participants, sought out her own form of support in online support groups for people with narcolepsy. These support groups proved to be invaluable for Bethany as she was able to receive emotional support which in turn helped her to feel less alone in her struggles:

*If I hadn’t found my support groups, I literally do not know where I would have been. I have cried on those like good gosh, Zoom meetings like to these people. I have not met any of them in person, but I have been talking to them for two years now. Yeah, like I, I just. I know some of them so well and I just. It’s so powerful to be able to go and talk to someone that gets it and can complete sentences*

**-*Bethany***

From feeling controlled by symptoms to feeling different to others, narcolepsy can be an extremely isolating experience. This is exacerbated by the fact that there appears to be a lack of support offered to participants. Feeling understood in their struggles is a valuable experience.

## Discussion

This study has explored the lived experience of being diagnosed with and living with narcolepsy. Three themes were the result of an Interpretative Phenomenological Analysis: minimising, dismissing and downplaying symptoms; navigating the winding journey to diagnosis; and a different way of living.

### Minimising, dismissing and downplaying symptoms

A noteworthy aspect of the narcolepsy experience is the minimisation of symptoms, particularly excessive daytime sleepiness. There were times when excessive daytime sleepiness occurred but it was not recognised as a symptom. Not noticing symptoms appeared to be the first stage of the illness experience which differs from the illness-constellation model (Moore and Johnson, 1991). Instead of the first stage being where individuals notice something is wrong, for narcolepsy, there may be a prior stage to this. Seeing symptoms in hindsight was evident, demonstrating that a considerable stage of the narcolepsy illness experience is a lack of symptom recognition.

Lack of symptom recognition appeared to stem from excessive daytime sleepiness being minimised, dismissed and even viewed as a ‘joke’. One consequence of this was the delay in individuals seeking help, with an average time of 8 years from symptom onset to first appointment, which is in line with previous research (Ohayon et al., 2021). Sleepiness was minimised as a unique but innate trait for some and considered part of their identity. For others it was minimised to be nothing more than a universal experience. Both responses draw attention to previous research that highlights the lack of awareness of the importance of sleep (Perry et al., 2013). Sleepiness was dismissed and minimised by doctors as being caused by hormones or vitamin deficiency. Doctors did not consider the sleepiness a symptom that required further investigation. Not considering sleepiness to be a symptom supports previous research that indicates that doctors have a lack of awareness of sleep disorders (Hassed et al., 2017; Saleem et al., 2017).

A novel finding from this study was that this minimising persisted *after* diagnosis. Participants downplayed and struggled with voicing the impact of narcolepsy on their lives: both stemming from and reinforcing the notion that sleep is not taken seriously. Consequently, the impact of narcolepsy is dismissed and underestimated, which participants seem to internalise as they feel that they have no right to complain. This is reflected further in how participants minimise their struggles and their feelings of guilt for accessing disability support that they are entitled to. Downplaying the impact of symptoms is thought-provoking when considered in the context of previous research; the impacts of narcolepsy are equivalent to or worse than the impact of Parkinson’s or epilepsy (Teixeira et al., 2004), and quality of life is much lower than people with other chronic diseases (Tadrous et al., 2021).

### Navigating the winding journey to diagnosis

As there is little qualitative research exploring the holistic experience of narcolepsy, one objective of the research was to capture the full illness experience. For our participants, a compelling part of the narcolepsy experience was navigating the journey to diagnosis. The first step in the journey involves noticing and acknowledging that something is wrong, which aligns with the first stage of the illness-constellation model (Morse and Johnson, 1991). However, for narcolepsy, this emerges as the second stage of the whole narcolepsy illness experience. The realisation that something was wrong came to participants when their sleepiness started impacting their social or academic lives. For others, their first experience of cataplexy was what led to their realisation. Participants’ experience of acknowledging that something was wrong but not knowing what was wrong further aligns with the first stages of the illness-constellation model (Morse and Johnson, 1991).

Arguably the most prominent part of navigating the journey to narcolepsy diagnosis is the second stage of the illness-constellation model: where individuals seek help and search for answers. This was a frustrating time for participants due to the extended amount of time spent searching for answers. The average time from first seeking help to diagnosis was 5 years. In consonance with previous research, part of this involved going back and forth to doctors (Kryger et al., 2002). Some individuals allowed the doctors to take control of their search for answers. In alignment with the biomedical model of health, participants considered doctors to be the experts of their health (Thorne, 2000). However, a remarkable challenge that individuals with narcolepsy face is the lack of knowledge from healthcare professionals. This lack of knowledge of narcolepsy among healthcare professionals corresponds with previous research into rare conditions (Budych et al., 2012). As a result, some participants received misdiagnoses that they intuitively knew they did not have, such as depression and functional neurological disorder. Thus, participants’ experiences were in line with previous research where they were treated for the wrong problem which delayed receiving a correct diagnosis and access to the correct treatment (Maski et al., 2017).

Other participants became proactive, searching for their own answers. As soon as individuals learned about narcolepsy, they intuitively knew that this was what was wrong with them. This suggests that the reason for not knowing what was wrong did not come from being unable to make sense of their symptoms. Instead, it was a lack of a label to put on the sense that they had made of their symptoms. Following this, participants experienced a change in attitude where they took a more proactive approach and asked doctors for referrals and sleep studies. However, these participants also faced issues that relate to the medical model of health: doctors prioritising their knowledge over their lived experience (Thorne, 2000). Consequently, participants faced the issue of doctors dominating the decision-making process in their health (Thorne, 2000).

In accordance with previous research, a fundamental part of participants’ experiences of narcolepsy experience was diagnostic delay (Khatami et al., 2016). The average time from symptom onset to diagnosis was 12 years which is similar to previous research (Frauscher et al., 2013; Ohayon et al., 2021). Considering the time gap between the previous research and this, the lack of awareness of sleep and sleep disorders is only accentuated. Yet this research provides insight into what influences this lack of awareness how participants live through this. Once participants had been referred to the sleep clinic, the process of receiving a diagnosis was quick. Participants experiencing a fast diagnostic process at the sleep clinic provide greater insight into participants’ experiences of diagnostic delays. Rather than undergoing a long process of several investigations and testing that is characteristic of other chronic health conditions such as rheumatoid arthritis (Majitha and Geraci, 2007), the experience of navigating the journey of a narcolepsy diagnosis is different. Participants spent a substantive amount of time trying to be taken seriously and negotiating a referral to the correct department. Ultimately, diagnostic delays are influenced by a lack of symptom recognition and sleepiness being dismissed or trivialised.

### A different way of living

Living with narcolepsy represented a different way of living for participants. As proposed by the illness-constellation model, individuals go through a stage of trying to make sense of the illness and adapting to the new level of functioning (Morse and Johnson, 1991). Interestingly, learning about narcolepsy helped participants to make sense of symptoms that they had been experiencing for many years prior to receiving a diagnosis but they struggled with accepting their new level of functioning. This resulted from the fact that living with narcolepsy affected several areas of participants’ lives (as per Goswami, 2016). They had to be aware of the impact of each activity on the next which often put limits on their social lives (Dodel et al., 2007; Teixeira et al., 2004) and involved strategic management of their day-to-day lives. They struggled with this since they felt different to their peers. This resulted in participants feeling controlled by their symptoms, even when taking medication, reinforcing the notion that pharmacological management of narcolepsy is insufficient (Maski et al., 2017). This is significant as an important aspect of living and adjusting to chronic illness is feeling that they have control over their illness (Hagger and Orbell, 2003). Interestingly, these experiences are not necessarily unique to narcolepsy as they have been evidenced with other conditions termed ‘Energy Limiting Chronic Illnesses’ (Hale et al., 2021). A defining feature of these is the fact that individuals must carefully consider what activities they can perform and that what has been done before plays a significant role in their ability to do tasks. Retheorising narcolepsy as not just as a sleep disorder, but as an Energy Limiting Chronic Illness may create fruitful avenues for future research.

Navigating these psychological and social changes has been evidenced for a long time as part of adjusting to living with chronic illness (Corbin and Strauss, 1991). Additionally, living with narcolepsy evidently had significant consequences for participants’ lives, which is associated with poor adjustment (Hagger and Orbell, 2003). Strikingly, what was evident in participants’ accounts was how they felt that they were left to do this alone. They recall the lack of support that they received following their diagnosis other than receiving medication. Having access to medication is important, but this alone is not sufficient. This lack of support is so consequential given the evidenced impact of narcolepsy psychosocially. Arguably, this results from the fact that narcolepsy, like other chronic illnesses, is primarily understood from a biomedical perspective (Hall, 1996) and not understood in the wider context of an Energy Limiting Chronic Illness (Hale et al., 2021).

This analysis has illuminated that what underpins the narcolepsy experience is a lack of awareness, knowledge and understanding of sleep and narcolepsy. These compounding factors contribute significantly to diagnostic delays, as individuals face not being taken seriously by others due to a lack of awareness of the importance of sleep. A lack of knowledge from doctors delayed the referral to the correct department, which delayed the diagnosis. Additionally, a lack of awareness of how significantly sleep disorders impact individuals’ lives results in a lack of support and research. Therefore, individuals’ symptoms are not recognised, and they struggle to get a diagnosis. Following this, individuals with narcolepsy rely on ineffective medication without adequate support.

### Strengths and limitations

This research was able to provide a novel in-depth insight into the whole experience of narcolepsy from symptom onset to management. IPA allowed us to explore how participants navigated their journey and made sense of their symptoms, the diagnosis process and living with narcolepsy. Mirroring a statement made by one participant (‘it’s hard for people to understand if you haven’t been through it’) the first researcher’s own experience of narcolepsy helped to gain an insider perspective; a ‘way in’ to authentically engaged with the narcolepsy experience.

However, it is important to note the limitations of this research. Threats to the homogeneity of the sample arise from the USA/UK mix of participants. Yet despite navigating different health systems, participants’ experiences were extremely similar. While a homogenous sample is important for IPA, this sample only consisted of females with type 1 narcolepsy. Type 1 narcolepsy has cataplexy, which was a significant aspect of the diagnostic journey for some participants. Therefore, the diagnosis experience may not be representative of the experience of type 2 narcolepsy. Additionally, the female experience of navigating a diagnosis may not be representative of the male experience.

### ‘Real world’ applications and future research

This research highlights that there is still a need for greater awareness of narcolepsy. In a broader sense, there is a need for greater awareness of sleep health and sleep issues in both the general population and medical professionals. This work highlights the need for greater support for people with narcolepsy. Initially, an easy way to adopt this could be to have a leaflet given to individuals upon diagnosis that signposts where they can find support; a suggestion given by one of the participants. Considerations of narcolepsy as an Energy Limiting Chronic Illness could help the development of an intervention to help individuals adjust to and manage to live with narcolepsy. The results of this study forming a underpinning: that it is less about achieving control of symptoms but more about gaining knowledge in how to strategically manage their energy limits.

Further research could benefit from multi-perspectival IPA studies with the inclusion of family members to explore their experiences and how they made sense of individuals with narcolepsy’s symptoms and diagnostic journey (Larkin et al., 2019). Future research would also benefit from a specific exploration into the minimisation of symptoms. Discourse analysis of individuals’ use of language might provide useful insights into how this ministration is achieved in everyday discussions; or how medical knowledge is operationalised to actively downplay the severity of symptoms. Such research would help to build on this study’s foundations in exploring how these dismissals impact help-seeking behaviour and health-related quality of life.

## Conclusions

In conclusion, this research study provides greater insight into the lived experience of narcolepsy. This study highlights how individuals navigate the process of making sense of their symptoms, the diagnosis journey and living with narcolepsy. This research further emphasises the need for increased awareness of sleep and sleep disorders.
